# Regulation of Rhodopsin-eGFP Distribution in Transgenic *Xenopus* Rod Outer Segments by Light

**DOI:** 10.1371/journal.pone.0080059

**Published:** 2013-11-15

**Authors:** Mohammad Haeri, Peter D. Calvert, Eduardo Solessio, Edward N. Pugh, Barry E. Knox

**Affiliations:** 1 Departments of Neuroscience and Physiology, Biochemistry and Molecular Biology, and Ophthalmology, SUNY Upstate Medical University, Syracuse, New York, United States of America; 2 Center for Neuroscience, University of California, Davis, California, United States of America; Doheny Eye Institute and Keck School of Medicine of the University of Southern California, United States of America

## Abstract

The rod outer segment (OS), comprised of tightly stacked disk membranes packed with rhodopsin, is in a dynamic equilibrium governed by a diurnal rhythm with newly synthesized membrane inserted at the OS base balancing membrane loss from the distal tip via disk shedding. Using transgenic *Xenopus* and live cell confocal imaging, we found OS axial variation of fluorescence intensity in cells expressing a fluorescently tagged rhodopsin transgene. There was a light synchronized fluctuation in intensity, with higher intensity in disks formed at night and lower intensity for those formed during the day. This fluctuation was absent in constant light or dark conditions. There was also a slow modulation of the overall expression level that was not synchronized with the lighting cycle or between cells in the same retina. The axial variations of other membrane-associated fluorescent proteins, eGFP-containing two geranylgeranyl acceptor sites and eGFP fused to the transmembrane domain of syntaxin, were greatly reduced or not detectable, respectively. In acutely light-adapted rods, an arrestin-eGFP fusion protein also exhibited axial variation. Both the light-sensitive Rho-eGFP and arrestin-eGFP banding were in phase with the previously characterized birefringence banding (Kaplan, Invest. Ophthalmol. Vis. Sci. 21, 395–402 1981). In contrast, endogenous rhodopsin did not exhibit such axial variation. Thus, there is an axial inhomogeneity in membrane composition or structure, detectable by the rhodopsin transgene density distribution and regulated by the light cycle, implying a light-regulated step for disk assembly in the OS. The impact of these results on the use of chimeric proteins with rhodopsin fused to fluorescent proteins at the carboxyl terminus is discussed.

## Introduction

**Figure 1:**
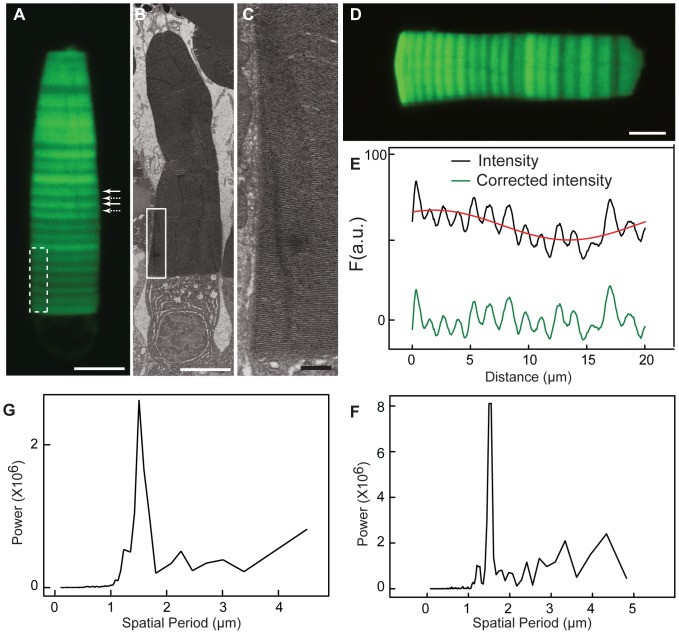
Periodic axial variation in fluorescence intensity in OS expressing a Rho-eGFP transgene. (A) The expression profile of OS (OS) demonstrates a varying level of Rho-eGFP in disk membranes along the OS axis in animals housed in a 24 h (12D:12L) cycle. Periodic axial variation is seen as alternating series of bright fluorescent regions (*solid arrows*) and dim regions (*dotted arrows*).(B, C) Electron micrographs of a rod photoreceptor, expressing Rho-eGFP, show equal spacing between disk membranes. Panel C is an enlargement of white box in panel B. The dotted white box in panel A illustrates a similar sized area for comparison. White bar is 5 µm and black bar is 200nm. (D, E) A rod expressing Rho-eGFP was analyzed to determine the intensity difference at peaks and troughs. The green trace represents the periodic variation in fluorescence intensity before (*black trace*) and after (*green trace*) subtraction of the aperiodic axial variation fit to a sinusoidal function (*red trace*). The amplitude of the variation between the maximum and minimum for each period was1.3±0.07 (mean ± S.D.) calculated from 90 periods taken from10 cells.(F) Fourier transform analysis of the proximal 20 µm of the OS shown in panel (B) demonstrates a peak at 1.5 µm representing the period of axial variation. The Fourier transform spectra are presented in terms of the spatial periods, and not as the more typical frequencies.(G) Fourier transform analysis of the proximal 20 µm of the OS averaged from five different cells also shows a period at 1.5 µm.

The vertebrate photoreceptor is a highly polarized neuron with a modified cilium specialized for light detection. The cilium contains an OS with a stack of hundreds of disks enclosed in the plasma membrane (Fig. 1) [1]. Rhodopsin is the major protein in the OS, comprising approximately 90% of the membrane protein complement [2]. New rhodopsin molecules are made in the ER, transported via a complex vesicular pathway to the base of the OS and inserted into new disk membranes [3]–[5]. Previously made disks then move apically and the oldest disks at the OS tip are shed and taken up via phagocytosis by retinal pigment epithelium. This disk renewal occurs every day [6], [7]. Accordingly, the whole length of the OS is renewed in 10 days for mammals and ∼4–6 weeks for frogs depending upon the temperature. Disk formation is stimulated by light [8], [9], but rhodopsin synthesis does not appear to be diurnal, at least in *Xenopus* [10]. Ultrastructural studies reveal a homogeneous distribution of disk membranes throughout the length of the OS [1]. However, light microscopy demonstrates inhomogeneities along the OS length, as birefringence bands perpendicular to the rod axis that arise from anisotropy in refractive index along the OS axis [11]–[14]. First observed in amphibian rods, they are also found in mammals [15]. In *Xenopus*, the birefringence banding pattern has a spatial period of about 1.0 –1.6 µm, which corresponds to ∼35 – 60 disks [16]. They are more pronounced at the base of the OS. The spatial periodicity is modulated by the length of the light-dark cycle, and is abolished under constant illumination conditions [17]. While these observations suggest an underlying variation in the disks produced in light compared to dark [18]–[20], the origin of the light-cycle dependent banding remains unexplained.

Imaging of fluorescently tagged proteins offers a powerful method for quantifying protein expression in photoreceptors [21]. In combination with systems for expression of transgenes in *Xenopus* photoreceptors [22], detailed measurements have been made on the distribution of soluble proteins [21], light-dependent protein movement into the OS [23], targeting signals necessary for rhodopsin OS localization and trafficking of membrane proteins to the OS [24]–[28] and diffusion of both soluble [29] and membrane-bound [30] proteins. Previously, a an eGFP tagged rhodopsin rhodopsin eGFP fusion protein (Rho-eGFP) was shown to exhibit non-uniform fluorescence intensity along the OS axis both in fixed [24] and live [31] samples, suggesting a time-varying production of the transgene. We employed confocal imaging in live rods [21], [31] to quantitate the Rho-eGFP distribution in the OS. We found that the variation in fluorescence intensity of this protein along the OS axis is coincident with the birefringence pattern and controlled by the light cycle. However, other integral membrane or membrane-associated fusion proteins, exhibit significantly reduced OS axial variation. Thus, these results suggest that there is a light-regulated pathway for trafficking membrane-associated proteins to the OS.

## Results

### Axial variation of Rho-eGFP distribution in *Xenopus* OS

The Rho-eGFP fusion protein binds 11-*cis* retinal, activates transducin and is transported predominantly to the OS [31]–[33]. We have previously reported that the expression levels of rhodopsin transgenes under control of the XOP promoter are substantially lower (<5%) than endogenous rhodopsin [31] and do not represent a significant overexpression of this membrane protein. However, the distribution of fluorescence in the OS is not spatially uniform exhibiting two types of axial variation. First, there is a prominent periodic axial variation that appears as a regular pattern of alternating bright (Fig 1A, solid arrows) and dim fluorescent segments (Fig. 1A, dotted arrows) perpendicular to the rod axis. Second, there is a gradual axial variation in the general expression level along the OS axis. This can be seen in the OS shown in Fig. 1A as relatively brighter apical fluorescence compared to the dimmer basal fluorescence. The variation can sometimes be extreme (Fig. S1) and is less pronounced in F1 and subsequent generations of transgenic lines (*data not shown*). This variation, which we term asynchronous variation, does not align between rods from the same retina and is related to mosaic transgene expression.

The OS ultrastructure in transgenic animals expressing Rho-eGFP had a uniform distribution of disk membranes (Fig. 1B and C) and we did not detect any periodic axial variation in morphology. To determine the spatial period in the fluorescence variation, we computed the discrete Fourier transform on OS regions within 20 µm proximal to the base, where banding is most regular and not distorted by OS stretching or swelling. An example cell is shown (Fig. 1D) with its intensity profile(Fig. 1E) and power spectrum (Fig. 1G). Note, the data are presented in terms of spatial periods, and not as the more typical frequencies, of the FT components to facilitate comparisons with axial displacement values (*see below*). We found a prominent peak in the power spectrum at a spatial period of 1.5 µm (Fig. 1F), which is equivalent to ∼54 disks [8]. The averaged power spectrum from 5 cells shows a similar peak at 1.5 µm (Fig. 1G). This spatial period is characteristic of periodic axial variation in all OS taken from animals housed in a 24 h (12D:12L) lighting cycle.

To determine the magnitude of the periodic axial variation, it was necessary to correct the OS fluorescence intensity for asynchronous variation in transgene expression. We found that in most but not all cells, the asynchronous variation could be fit by a sinusoidal function (Fig. 1E and Fig. S2). Unlike the periodic axial variation, there was a wide range of spatial periods, with a median of 27 µm, which is roughly half the length of the OS in animals housed in a 24 h (12D:12L) lighting cycle. Images were deconvolved to compensate for the PSF blurring. The ratio of corrected fluorescence intensity of peak to adjacent trough was 1.3±0.07 (N = 90 peaks from 10 cells). These results indicate ∼30% higher Rho-eGFP density in regions of maximal fluorescence intensity compared to adjacent regions with minimal local intensity.

**Figure 2:**
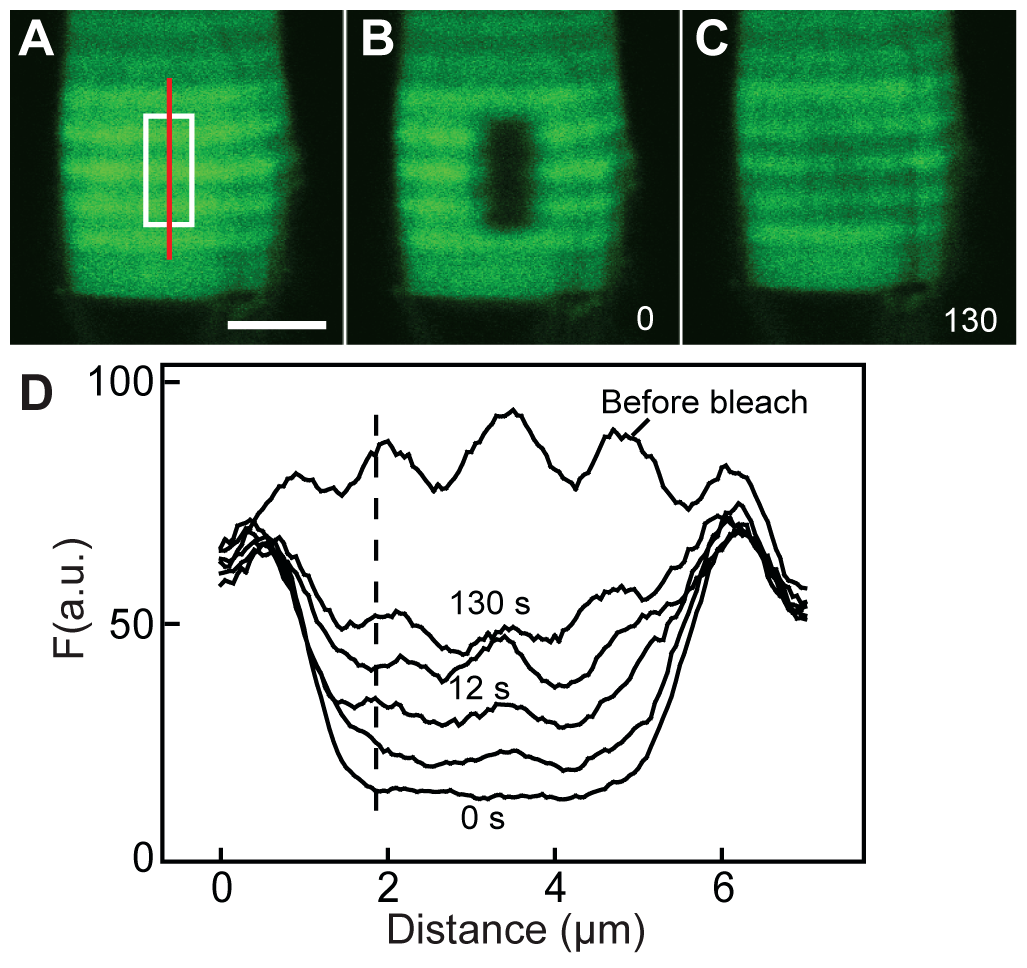
Fluorescence recovery after photobleaching (FRAP) in a live rod expressing Rho-eGFP. (A–C) Sequential fluorescent images from a cell are shown before (A), immediately after photobleaching (B) and 130 s later (C). The target area is highlighted in A with a white box.(D) The fluorescence intensity profile scanned through the photobleached area (red line in panel A) demonstrates the recovery of the banding pattern in register with the non-bleached neighboring area.

To determine whether Rho-eGFP found in the periodic bands are free to move laterally in the disk membrane, fluorescence recovery after photobleaching was used. Following the localized photobleaching across several bands of Rho-eGFP density, fluorescence intensity recovered substantially and stabilized after 130 s. Moreover, the recovered region remained in alignment with bands of similar intensity in neighboring non-bleached area (Fig. 2). Thus, consistent with the expected insertion of the Rho-eGFP transgene into discrete disc membranes, fluorescence redistribution after photobleaching was restricted to lateral mobility.

**Figure 3:**
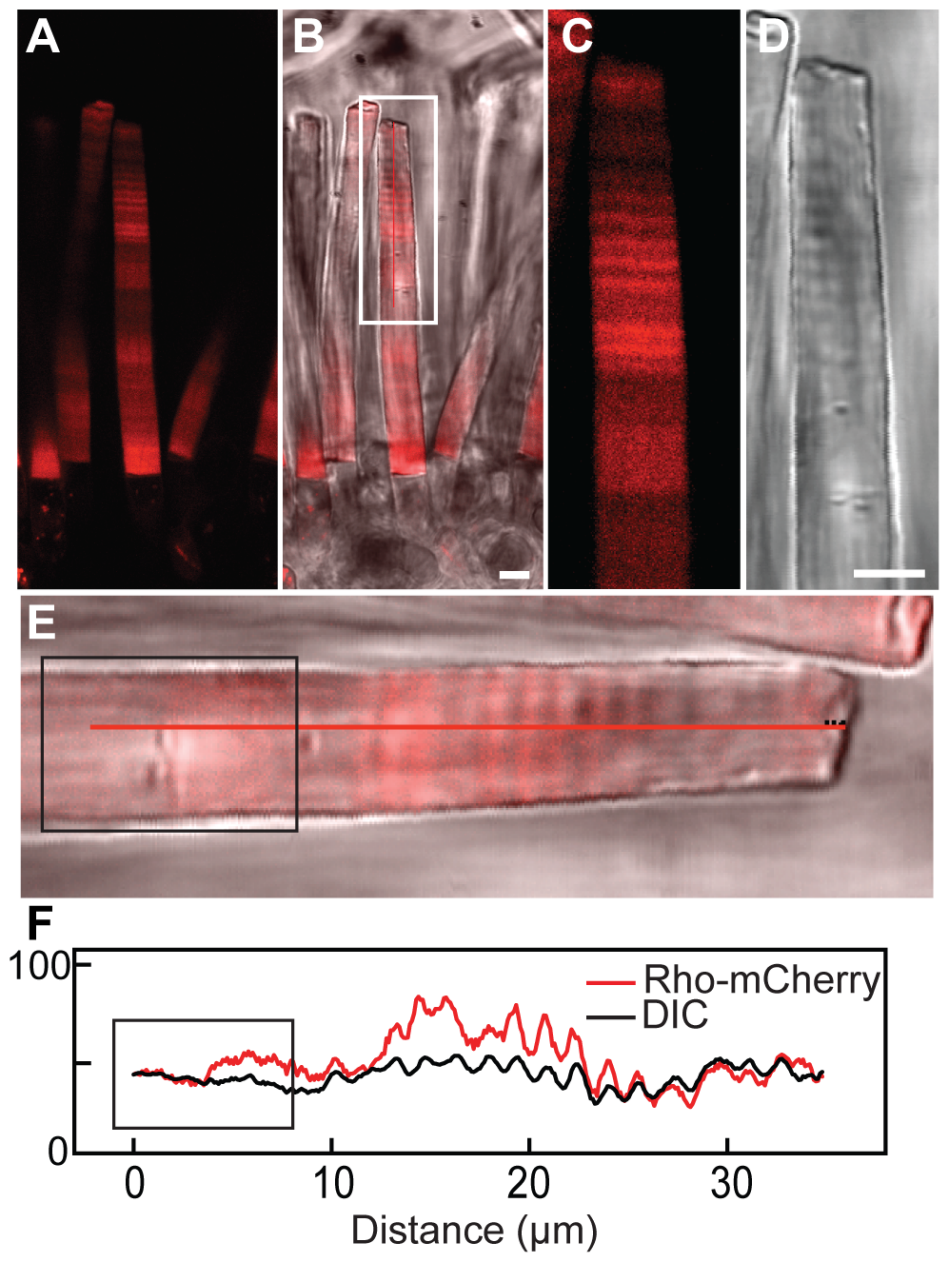
Comparison of periodic axial transgene fluorescence and refractive index variation measured by interferometry. (A–D) The fluorescent and DIC images of a live retinal chip (*white box*, enlargement of a region of an OS shown in C, D) expressing Rho-mCherry exhibit similar periodic banding in both preparations. (E, F) The intensity profile (F) of the fluorescent and DIC images for rod shown in E demonstrates a synchronized banding pattern. The animal from which the rod shown in E was taken was moved from a 24 h (12D:12L) cycle into constant light (approximate region is boxed in *black*) for over a week before sacrifice. White bar is 5 µm.

The periodic axial variation was also observed in animals expressing a Rho-mCherry transgene (Fig. 3A boxed region from the 24 h (12D:12L) lighting cycle period). The periodic axial variation of Rho-mCherry and Rho-eGFP was coincident in rods co-expressing both transgenes, although the asynchronous variation was not as tightly linked (Fig. S3A). We also compared periodic axial variation to the birefringence banding previously studied by Kaplan [17]. We obtained DIC images and fluorescence intensities from rods expressing Rho-mCherry (Fig. 3C–3F). In this example, the animal had been housed in a 24 h (12D:12L) lighting cycle for several weeks and then switched to constant light for a week (Fig. 3E, boxed region) prior to imaging. In the 24 h lighting cycle, Rho-mCherry fluorescence intensity was closely correlated with the alternating dark-light bands in the DIC image (Fig. 3F). However, when the animal was housed in constant light, which abolishes birefringence banding [17], the Rho-mCherry periodic axial variation was eliminated (Fig. 3E, boxed region). Thus, since birefringence banding is strongly regulated by the light cycle [17], we examined the periodic axial variation of rhodopsin transgenes in rods from animals housed in different lighting cycles.

### The light cycle rather than circadian factors, regulates axial variation of Rho-eGFP

**Figure 4:**
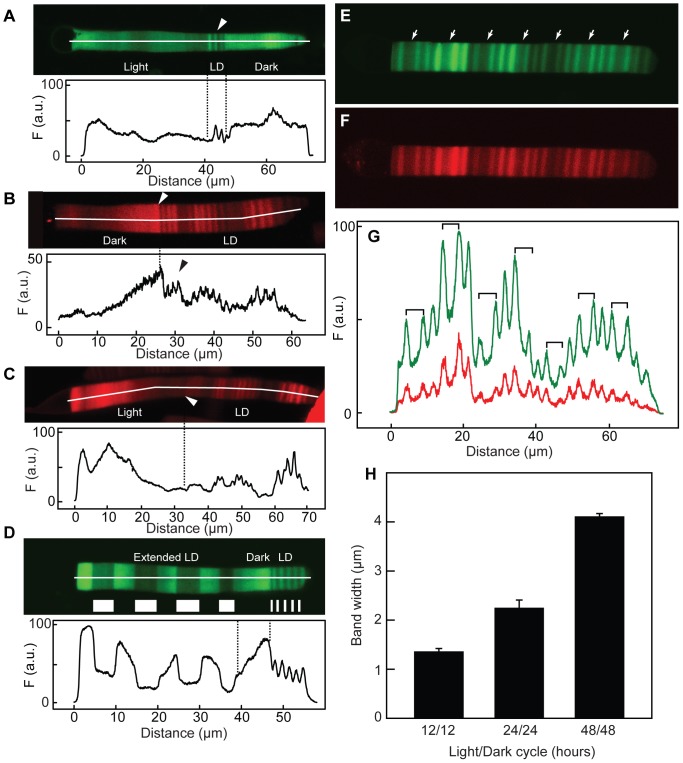
Effect of varying light-dark cycle on periodic axial variation. (A) Transgenic animals expressing Rho-eGFP were housed for 2 weeks in constant darkness, switched to 24 h (12D:12L) for 3 days (LD) and then in constant light for 2 weeks prior to imaging. The intensity profile of the cell is shown below. While axial banding with spatial period 1-1.4 µm are seen in the 24 h light cycle (*arrow*), little variation with this spatial period was observed in constant dark or light. Note that the rate of membrane addition to the OS is higher in the light than in either cycling or constant dark conditions. (B) Transgenic animals expressing Rho-mCherry were housed in 24 h (12D:12L) light cycle for over 4 weeks (LD) and switched (approximate position indicated by the *arrow*) to constant darkness for over 2 weeks before imaging. Axial banding with spatial period 1–1.4 µm is seen in the 24 h light cycle (*arrow*), no variation with this period was observed in constant dark. The intensity profile of the cell is shown below. (C) Transgenic animals expressing Rho-mCherry were housed in 24 h (12D:12L) cycle for over 4 weeks (LD) and switched (approximate position indicated by the arrow) to constant light for over 2 weeks before imaging. Axial banding with spatial period 1–1.4 µm is seen in the 24 h light cycle (LD), no variation with this period was observed in constant dark. The intensity profile of the cell is shown below. (D) Transgenic animals were first housed in a 24 h (12D:12L) cycle for over 4 weeks, switched to constant dark for 7 days and then maintained in a 168 h (84D:84L) cycle until imaging. The animals were sacrificed during the dark period. The approximate regions synthesized in different lighting periods (brighter regions synthesized in the dark) are indicated. A spatial period of 4–6 µm was observed in the extended lighting cycle. The intensity profile of the cell is shown below. (E–G) Transgenic animals expressing Rho-mCherry and Rho-eGFP simultaneously were housed in an asymmetric cycle of 144 h ((24D:24L)_4_:48L) for 6 weeks. There were two different widths of bands assembled in the light, one with ∼2 µm and a wider one (arrows) with ∼4 µm. These are associated with the 24 h and 48 h light periods, respectively. (H) Frogs were kept in 24 h (12D:12L) cycle for 4 weeks and then moved to an asymmetric cycle of 96 h (24L:24D:24L:24D:48L). The widths of dark bands (error bars are SD) in cells (N = 3) from these animals were determined. Each light cycle width is statistically different from the others (p<0.5).

When transgenic *Xenopus* were switched from a 24 h (12D:12L) lighting cycle to constant dark or light, Kaplan banding disappeared in regions synthesized under the constant conditions (Fig. 4A–C). These data indicate that the periodic axial banding is under control of the light cycle rather than a circadian clock. Moreover, extended light cycles produced axial variation with different periodicities of alternating fluorescence intensity. For example, animals housed in a 168 h (84D:84L) cycle exhibited a much larger spatial period than those housed in 24 h cycle (Fig. 4D). The spatial period of the axial variation from three sets of animals housed in different conditions was 1.3 µm for a 24 h (12D:12L) cycle, 2.2 µm for a 48 h (24D:24L) cycle and 4.1 µm for a 96 h (48D:48L) cycle (Fig. 4H). The average magnitude of the maximal variation in fluorescence intensity in the 168 h (84D:84L) lighting cycle was 3-fold (N = 30 peaks taken from 10 cells).The relative widths of the Rho-eGFP bands synthesized in the light were larger than those in the dark, most easily appreciated in the extended lighting cycles (e.g. Fig. 4D–G).This is consistent with previous studies that showed a higher rate of disk synthesis in the light than the dark [7]. For animals housed in extended light cycles and sacrificed at the end of the dark period (for example, Fig. 4D), we found bright fluorescence in the most recently synthesized disks, suggesting that the bright fluorescence bands are synthesized in the dark. Similarly, animals sacrificed at the end of the light period had regions of lower fluorescence intensity at the base of the OS (*data not shown*). To confirm the period during which the different intensity bands are synthesized, transgenic frogs expressing Rho-eGFP and Rho-mCherry simultaneously were housed in an asymmetric cycle of 168 h ((24D:24L)_4_:48L) for 6 weeks (Fig. 4E–G). The fluorescent pattern had two cycles of axial variation whose widths were similar to animals housed in 48 h (24D: 24L) cycle followed by a low fluorescence region (Fig. 4E arrows) whose width was similar to animals housed in a 96 h (48D:48L) cycle. Coincident fluorescent patterns were observed for both transgenes (Fig. 4E and F). These results show that the regions of lower fluorescence intensity (and hence Rho-eGFP and Rho-mCherry density) were synthesized in the light while the regions of higher fluorescence intensity were synthesized in the dark.

There was a transition between the OS zone with periodic axial banding and aperiodic fluorescence variation in animals that transitioned from constant conditions into 24 h (12D:12L) cycling conditions (Fig. 4A), or from cycling conditions into constant dark (Fig. 4B and 4D) or light (Fig. 4A and 4C) indicating that the light cycle is directly responsible for regulating the periodic axial variation. Moreover, animals maintained in constant light formed longer OS, as previously described [34]. The light cycle did not influence the asynchronous variation (Fig. 4A–C).

**Figure 5:**
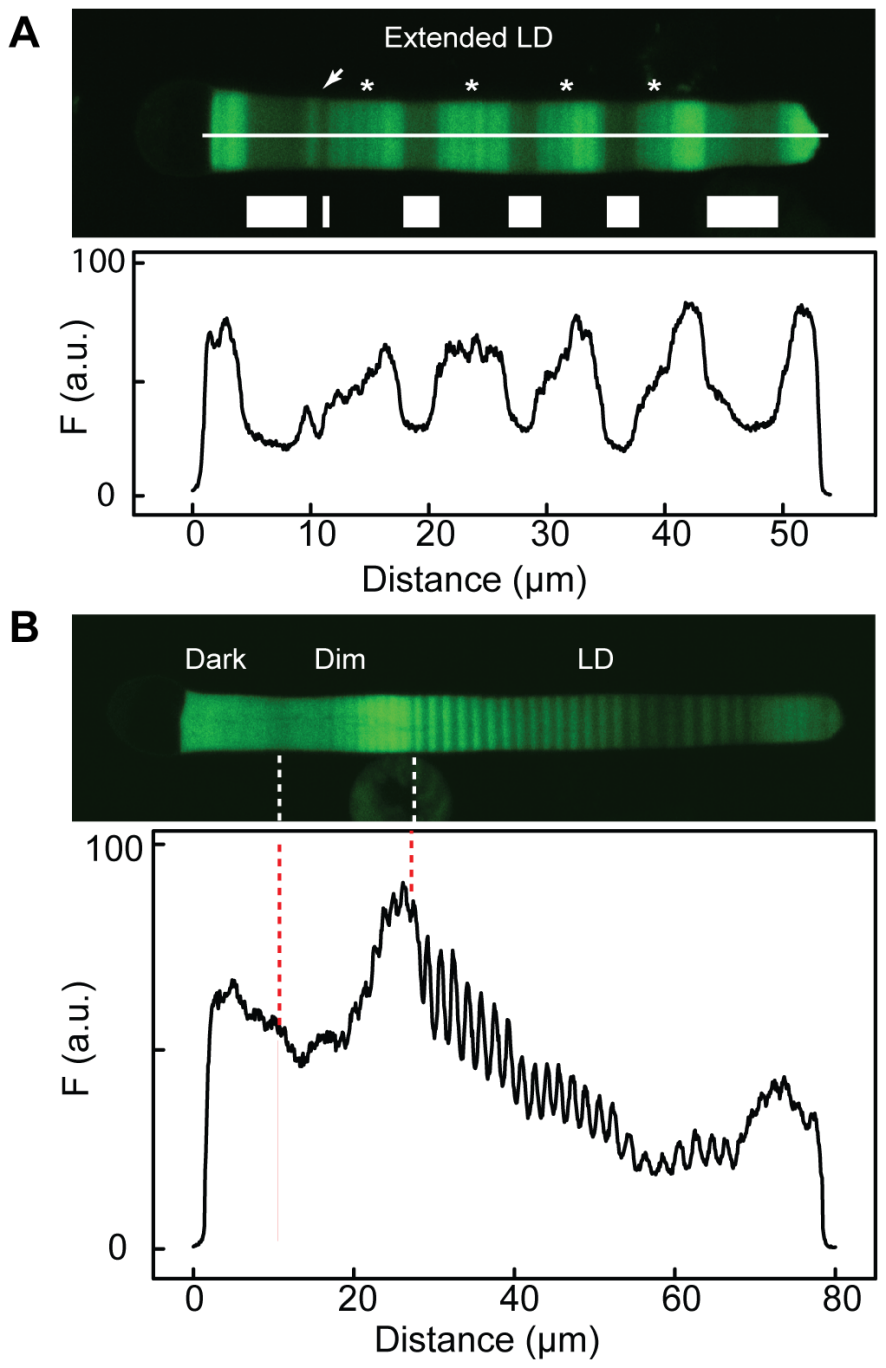
Periodic axial variation in dim light cycles. (A) Transgenic frogs expressing Rho-eGFP were kept for six weeks in 168 h (120D:48L) cycle. During the dark periods, there was dim light exposure since the incubator was not completely light-sealed. In these dark periods (asterisks), there was an increase in fluorescent intensity and an additional axial variation superimposed, with a spatial period of 4–6 µm. At the arrow, the animals experienced one 24 h (12D:12L) cycle and followed by 4 days light and finally a 3-day dark period. Animals were sacrificed during the dark period. The intensity profile of the cell is shown below. (B) Transgenic frogs expressing Rho-eGFP were kept first in a 24 h (12D:12L) cycle (LD) and then moved to an incubator was not completely light-sealed(Dim) for two weeks and were moved to a totally light-sealed chamber (Dark). Cells (N = 23) were imaged and a representative cell is shown. The light-sealed chamber did exhibit periodic axial banding as found in the other two regions. The approximate locations of the transitions in lighting are indicated. The intensity profile of the cell is shown below.

The periodic axial variation of Rho transgene density was very sensitive to light, and banding could be observed if animal housing was not completely light tight. In rods from animals housed in an extended cycle, axial banding from small light leaks can still be seen during the dark period (Fig. 5A, asterisks). In another experiment (Fig. 5B), transgenic frogs expressing Rho-eGFP were housed in a 24 h (12D:12L) light cycle, then moved to a darkened chamber for two weeks and finally into a totally light-sealed chamber for two weeks (Fig. 5B). In the first two periods, there was periodic axial banding with the expected spatial period of 1.0–1.5 µm. However, in the final period in complete darkness, no banding was detected. The magnitude of the amplitude of the axial variation appeared to increase when brighter lights were used (e.g. compare the amplitude in normal light versus in the dim light), but the asynchronous variation precluded reliable quantification.

### Axial density variation of endogenous rhodopsin

**Figure 6:**
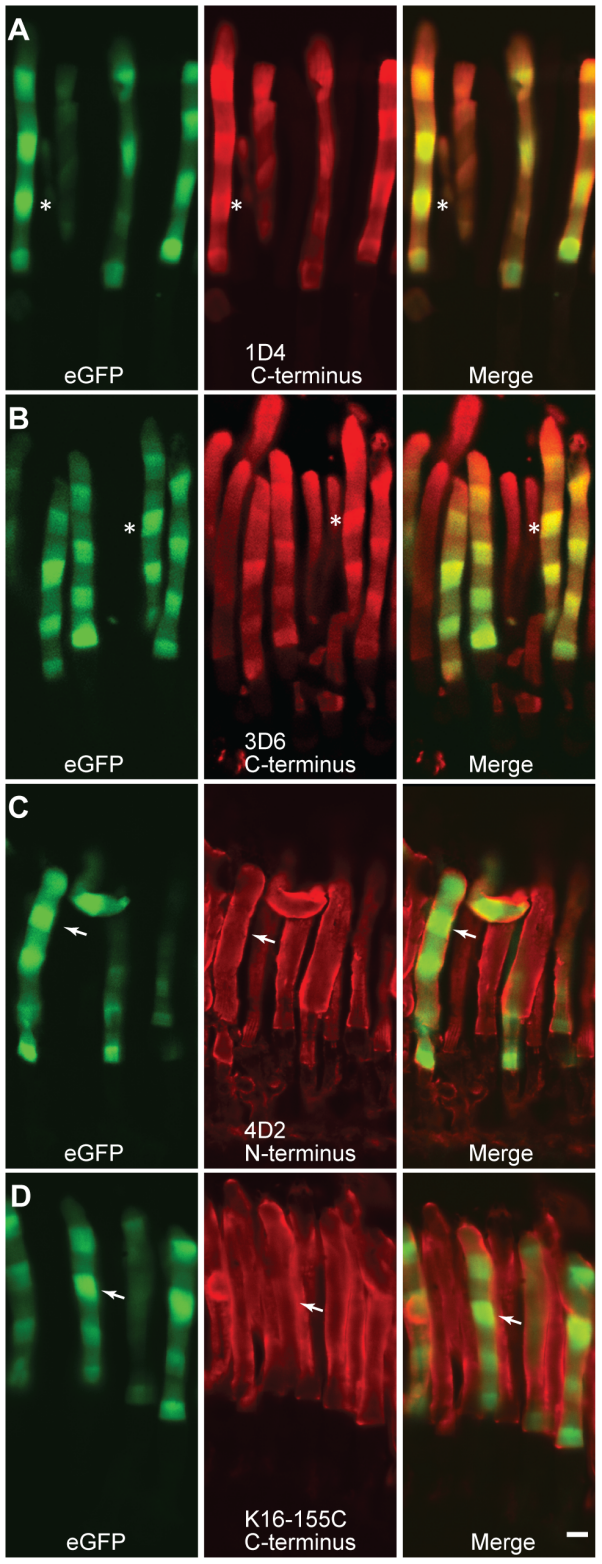
Immunostaining of transgenic retina expressing Rho-eGFP distributed in axial bands with anti-rhodopsin antibodies. Adult transgenic frogs expressing Rho-eGFP, which has been modified to contain an epitope for the monoclonal antibody 1D4, were kept for 8 weeks in a 168 h (84L–84D) light cycle and then eyes were fixed and immunostained with the indicated antibodies. Rho-eGFP signal (green) is the intrinsic from eGFP fluorescence. The antibodies were detected using a secondary antibody labeled with Cy3 (red). Confocal microscope images of individual and merged channels are presented. Examples of corresponding regions with high Rho-eGFP fluorescence are indicated in A and B by asterisks and in C and D by arrows. White bar is 5 µm.

We investigated whether endogenous rhodopsin exhibits periodic axial variation in OS density similar to that of fluorescent rhodopsin transgenes using immunohistochemistry and rhodopsin densitometry. To improve spatial resolution via increased band width and axial variation amplitude as predicted by Rho-eGFP, we housed animals in an extended168 h (84D:84L) cycle prior to sacrifice. We used antibodies that recognize the Rho-eGFP transgene alone via its C-terminal tag (1D4 and 3D6, [35]), antibodies that recognize endogenous Rho but not Rho-eGFP(K16-155C, [36]) and antibodies that recognize both endogenous and Rho-eGFP (4D2, [35]). Since the expression levels of Rho-eGFP are much lower than endogenous rhodopsin [30], [31], the 4D2 antibody will primarily report the endogenous rhodopsin. Note that the C-terminal antibodies had better penetration into the OS than the N-terminal antibody (Fig. 6). Bands of high Rho-eGFP fluorescence were detected with C-terminal antibodies that recognize the transgene (Fig. 6A and B, for example asterisks). However, there was no correspondence (Fig. 6C and D, for example *arrows*) between bands with high Rho-eGFP fluorescence and bands of higher reactivity with N-terminal antibodies that recognize endogenous rhodopsin. These micrographs also show that the antibodies have some penetration into the OS and also show that there is little fluorescence bleed-through in the Cy3 channel. With an antibody that specifically recognizes Rho-eGFP, the expected axial banding pattern was detected although the magnitude of the fluorescence variation between regions synthesized in the dark and light are somewhat reduced (Fig. 6). We attribute this reduction to alterations in cellular morphology caused by fixation. Given the ∼3-fold amplitude in Kaplan banding intensity of Rho-eGFP, we expect to be able to detect axial variation even at the margins of the OS (Fig. 6C and D). Thus, the endogenous and transgene rhodopsin axial distribution patterns were different.

**Figure 7:**
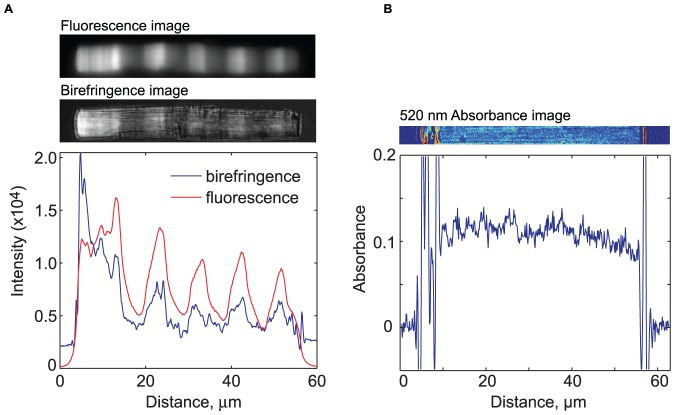
Axial rhodopsin absorbance is invariant despite large variation in Rho-EGFP levels. (A) A fluorescence image (top) and birefringence image (middle) of an outer segment of a Rho-EGFP–expressing rod from an animal housed on an extended 168 h (84D:84L) cycle. The intensity of the fluorescence and birefringence are plotted (bottom). (B) A 520-nm absorbance image (top) and the axial fluorescence profile (bottom).

We directly measured rhodopsin pigment concentrations using light-dark difference densitometry at 520 nm, which can reliably detect variations down to approximately 5% [30]. To amplify potential variations, we utilized rods derived from animals housed in an extended 168 h (84D:84L) cycle prior to sacrifice. Although the Rho-eGFP density exhibits ∼3-fold variation between the dark and light periods, there is no apparent variation in endogenous rhodopsin density (Fig. 7). We conclude that endogenous rhodopsin does not exhibit axial variation with the same amplitude as Rho-eGFP transgenes although we cannot rule out small changes below our limits of detection. Thus, it appears that Rho-eGFP is reporting the light-sensitive regulation of membrane assembly that is apparently not tightly coupled to transport of rhodopsin to the OS.

**Figure 8:**
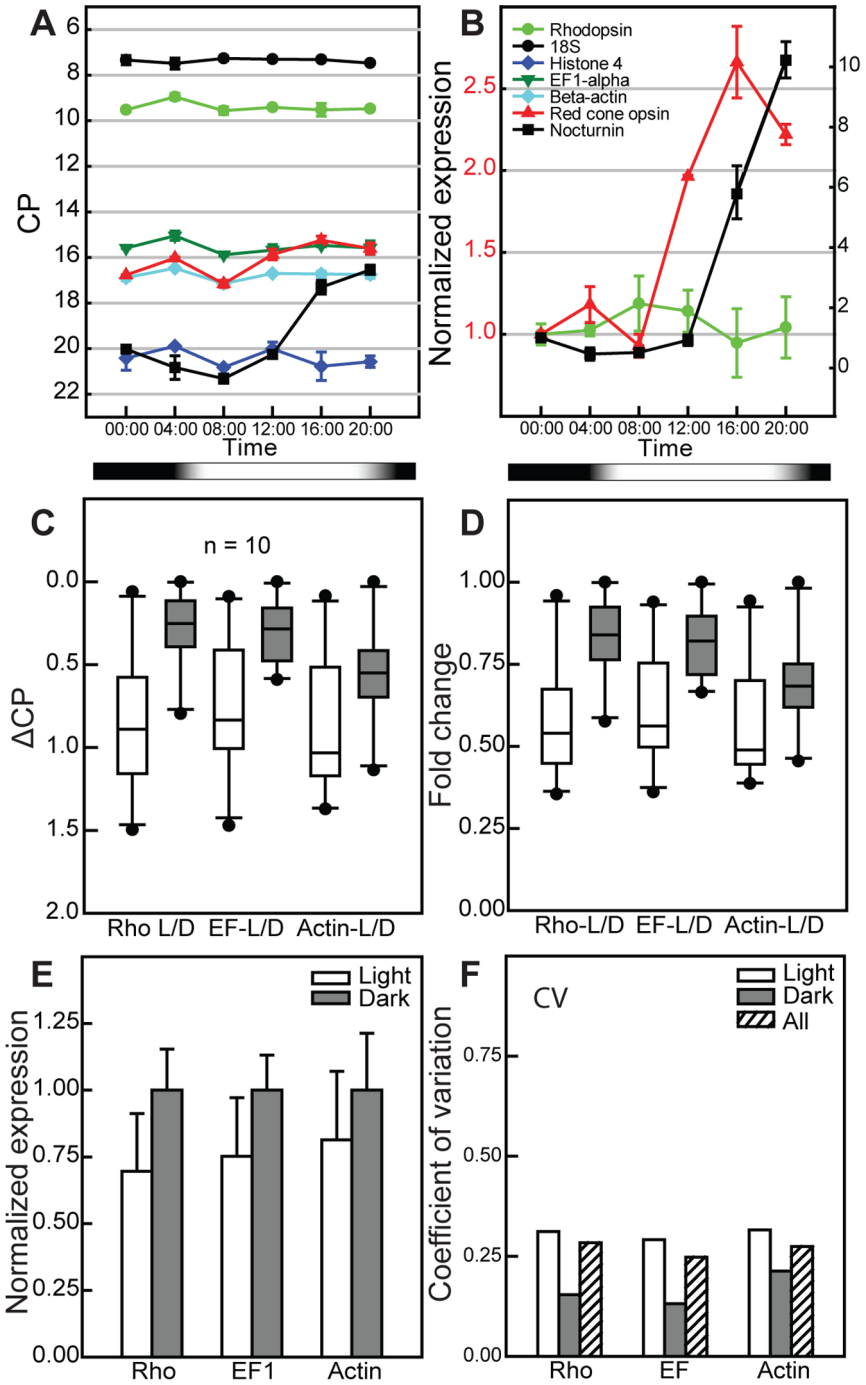
Expression level of rhodopsin transcripts at points in the light cycle. (A) Non-transgenic frogs were kept in 24 h (10D:14L) cycle and sacrificed at 4:00 hr time intervals and RNA extracted from eyes. Real-time PCR was used to quantify transcript levels using (A) PCR crossing point (CP) values. There is a slight increase in the transcript levels of rhodopsin, red cone opsin, and control genes, such as EF1-α and β-actin during the end of dark cycle. Black/white bar shows the dark/light periods (B) CP values normalized to housekeeping genes (*see methods for details*). The expression levels of rhodopsin and red cone opsin use the left axis scale while nocturnin uses the right Y-axis. The expression levels of rhodopsin, red cone opsin and nocturnin are shown. The expression of red cone opsin increased shortly after light onset, peaked a few hours afterwards and dropped in the dark. The expression of nocturnin increased before dark onset and peaked during the dark period and then returned to baseline. The expression level of rhodopsin, however, did not change significantly. (C, D) The expression levels of rhodopsin transcript and two control genes, EF1-α and β-actin, were determined during both a 24 hr light and 24 h dark cycle. The ΔCP (C) and fold change (D) in the light (L) and dark (D) values are shown as box plots. There is a higher expression level of all three genes during the dark cycle. The variation of the gene expression is much less during the dark cycle. (E) Expression levels normalized to 18S show ∼30% increase in all three genes during the dark cycle. (F) The coefficient of variation in the expression level of genes is shown during the dark or light cycle and combined (All).

We examined the expression level of selected transcripts in animals maintained in a 24 h (12D:12L) cycle using qRT-PCR to characterize changes in transcription throughout the light cycle. While both red cone opsin and nocturnin exhibited robust changes over 24 h (Fig. 8A and B), as previously reported [37], [38], endogenous Rho did not show a significant change (Univariate ANOVA, F[6], [7] = X, P<0.76, Fig. 8). We also did not find significant changes in the expression level of endogenous rhodopsin or Rho-transgenes in animals kept in 24 h light or dark (Fig. 8). However, since rhodopsin transgenes exhibit spatial variegation across the retina and also asynchronous (temporal) variation, whole retinal analysis of transgene transcripts is not definitive.

We next examined the OS distribution of Rho transgenes controlled by several other rod-specific promoters, including rod transducin alpha subunit and rod arrestin, and found identical periodic axial variation as that in animals utilizing the rhodopsin promoter (Fig. S3). Thus, while we cannot rule out regulation of Rho transgene transcription by light, the reproducibility of the spatial period and magnitude support an argument for a post-transcriptional mechanism to explain periodic axial variation.

### Banding pattern of membrane-associated fluorescent proteins

**Figure 9:**
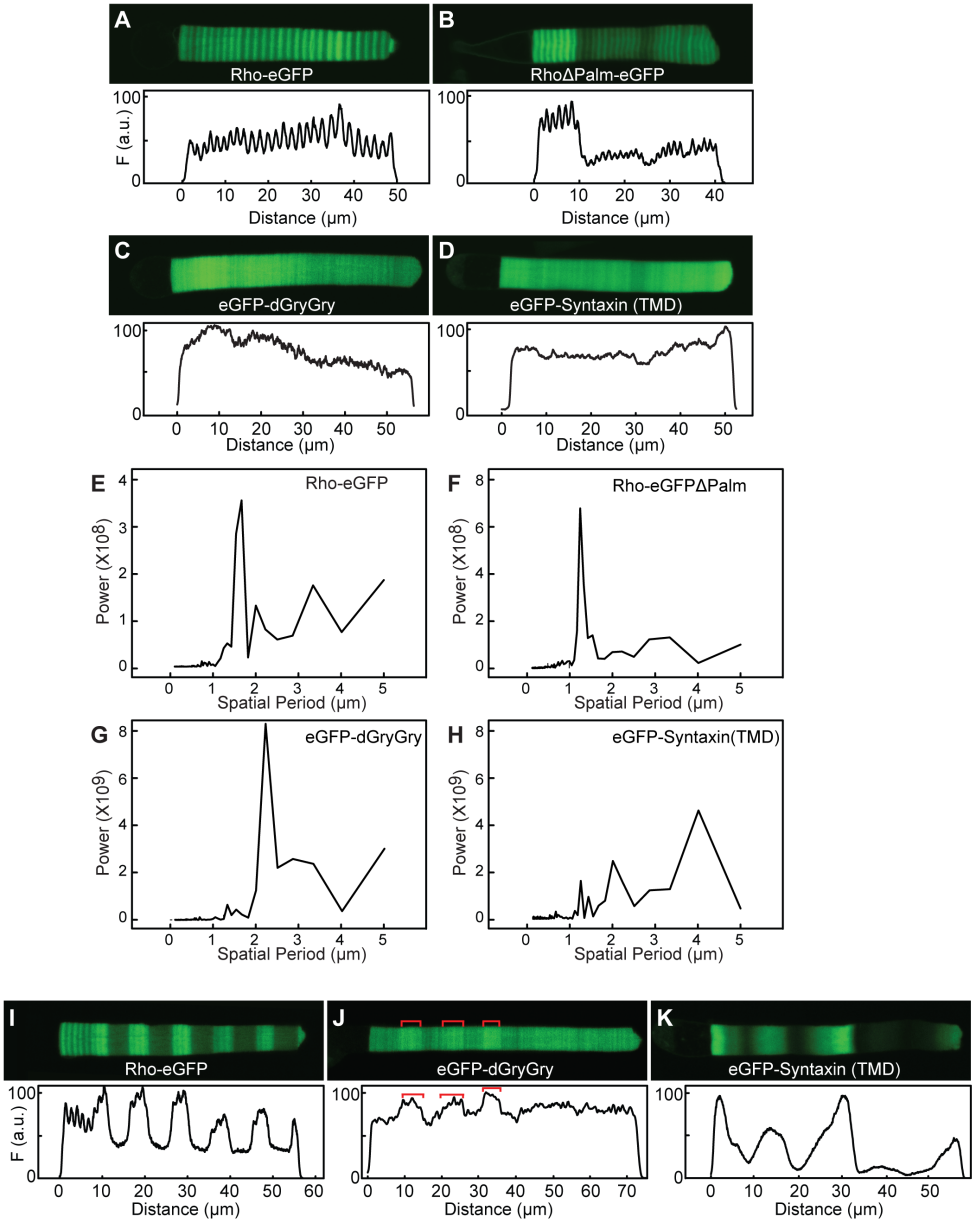
Axial variation of rhodopsin C-terminal mutants and membrane associated eGFP transgenes. Transgenic animals expressing the indicated transgene were housed in either a 24(12D:12L, A–D) or 168 h (84L:84D, I–K) light cycle. Representative cells and intensity profilesfrom animals housed in a 24 h (12D:12L) are shown. (E–H) Power spectra of the axial fluorescence intensity distribution from cells expressing the indicated transgenes. To enable comparisons of the power between different cells, the total OS fluorescence intensity for each cell was set to 1 and then the fluorescence intensity distribution was normalized to that value. Note the scales in E and F are different than G and H. RhoΔPalm-eGFP has the palmitoylation sites deleted (C322S and C323T), eGFP-dGyrGy contains two geranylgeranyl acceptor sites and eGFP-Syntaxin(TMD) has eGFP fused with the transmembrane domain from syntaxin.

To further explore the light-sensitive regulation of membrane protein distribution in the OS we expressed two other membrane or membrane-associated proteins under control of the XOP promoter: an eGFP with two protein prenylation signals at the C-terminus, resulting in the attachment of two geranylgeranyl (C_20_) moietie sand an eGFP fused to the syntaxin-3 transmembrane[26]. We compared the OS fluorescence intensity distribution to Rho-eGFP and RhoΔPalm-eGFP, in which two highly conserved vicinal cysteines (C322–C323) in the C-terminus were changed to serine and threonine, respectively. These mutations removed the two palmitoylation sites that anchor a portion of the C-terminus to the disk membrane surface. The RhoΔPalm-eGFP distribution had an axial variation similar in magnitude (compare Fig. 9A and B) and spatial period (compare 9E and F) as found in Rho-eGFP. Both Rho-dGryGry and eGFP-Syntaxin(TMD) were found predominantly in the OS (Fig. 9C and D), but there was significantly reduced periodic axial variation compared to Rho transgenes (Fig. 9A and B).For eGFP-dGryGry, the spatial period was significantly higher (2.3 µm) than for Rho transgenes. There is much less power (∼20%) in the normalized spectra at this spatial period than in Rho-eGFP. eGFP-Syntaxin(TMD) had very weak banding, with the major peak occurring at 4 µm, substantially higher than for Rho-eGFP. There was very little power (<5%) in spatial periods associated with the light-sensitive variation. Even in lighting cycles with extended dark and light durations, the magnitudes of periodic axial variation were significantly reduced for both dGryGry and eGFP-Syntaxin(TMD) (Fig. 9).

### Axial variation of Arrestin-eGFP distribution in light-adapted OS

**Figure 10:**
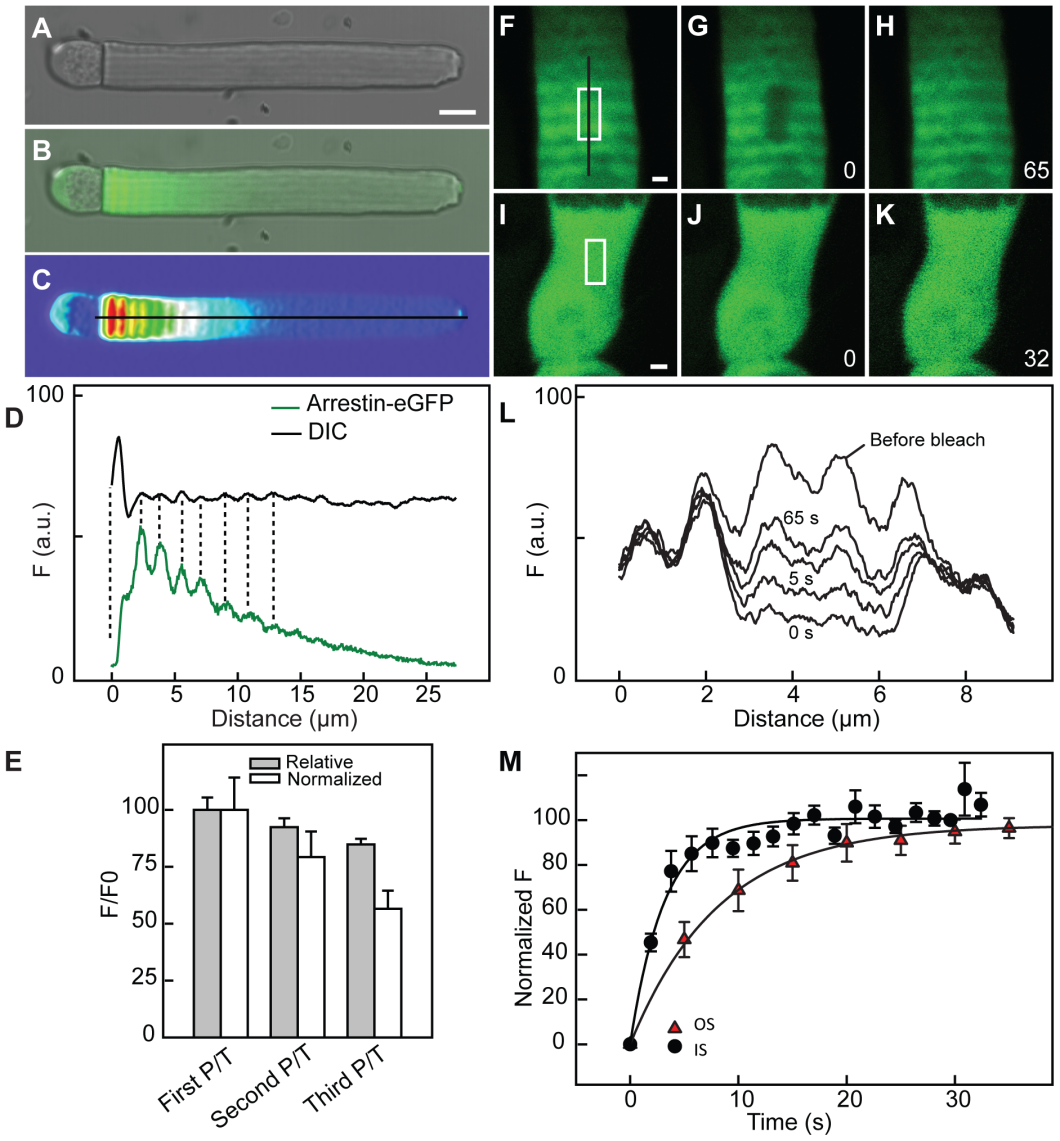
Axial variation of arrestin-eGFP transgenes in the OS. (A–E) A retinal explant from transgenic frogs expressing soluble arrestin-eGFP in rods was dark adapted and then moved to light for an hour before the live imaging. Arrestin-eGFP demonstrated axial variation in fluorescence at the base of the OS with a period of 1–1.4 µm that was synchronized with the DIC variation. The intensity profiles of the cell for both variations are shown (D). The intensity of the fluorescence decreased along the OS and the amplitude of the variation between peak (P) and neighboring trough (T) also decreased. The first peak was ∼35% brighter than the next trough (N = 5 cells, p<0.03). Peak/trough ratios (P/T) were calculated for the first three light cycles closest to the base and then normalized to the first peak which was set 100% (Relative) and also after normalization to the mean (Normalized). Error bars represent standard deviation and each of the three bands were statistically different (N = 5 cells, ANOVA p<0.0003). (F–M) Fluorescence recovery after photobleaching. The light adapted rod with arrestin-eGFP in the OS (F–H, L) and IS (I–K) was photobleached within an area near the base of the OS (*white box*) in panels (F, I) and the fluorescence recovery of the photobleached area was recorded continuously. The intensity profile of this recovery along the black line in panel (F) is shown in panel (L) as a function of time. There is recovery after photobleaching within 65 seconds in the OS, regenerating the axial banding pattern with the same spatial period as neighboring regions. The IS arrestin-eGFP recovered faster that that in the OS. White bars are 1 µm.

The birefringence and Rho transgene periodic axial variation suggest that there may be associated axial variation in other disk membrane properties. Arrestin-eGFP [21] binds to rhodopsin in a light-dependent fashion and undergoes light-dependent translocation to the OS[39]. Transgenic rods expressing arrestin-eGFP (eGFP at the C-terminus of arrestin) were light-adapted and OS fluorescence intensity was determined in live rods at various times after light exposure (Fig. 10). The OS distribution of arrestin-eGFP exhibited a similar periodic axial variation pattern as observed with Rho transgenes and was in phase with the birefringence banding pattern. Following photobleaching, the fluorescence recovered after 65 s to reproduce the pre-bleach periodic axial variation in alignment with bands in neighboring non-bleached area. We measured the rate of recovery of arrestin-eGFP in the IS and OS and found that the half-time of recovery for arrestin-eGFP in the OS is longer than that of the inner segment. Thus, arrestin mobility is primarily lateral, revealing it to be bound to rhodopsin or to another component of the disc membranes.

## Discussion

### Two types of variation in rhodopsin transgene expression

Using transgenic *Xenopus* and live cell confocal imaging, we have exploited the rod OS to examine membrane synthesis over many weeks. We have observed two types of variation in rhodopsin transgene density along the OS axis. One type is a slow modulation of the overall expression level that was not synchronized with the light cycle or between cells in the same retina. This variation is likely related to transgenic effects such as position effects (e.g. [40]) and/or epigenetic modifications of the transgene locus (e.g. [41]). However, this is this first time to the best of our knowledge that such slow temporal variation in transgene expression has been reported. The second type is a light-sensitive fluctuation between higher densities of rhodopsin fusion protein in membranes synthesized at night and lower densities in membranes synthesized during the day. The periodic axial variation disappears in constant light or dark conditions. The axial density distributions of rhodopsin fusion protein exhibited a spatial period of 1–1.5 µm in 24 h (12L:12D) lighting cycles at 18–20°C, which is very similar to the spatial period for birefringence banding [16] and to the daily increase in OS length [8].Because of the common properties shared between the periodic axial variation of Rho-eGFP/Rho-mCherry and the birefringence axial, we will refer to them both as Kaplan banding. Another transmembrane protein, peripherin-eGFP, appears to also exhibit Kaplan banding[28]. By contrast, the magnitude of the Kaplan banding was significantly reduced with other membrane-associated fusion proteins such as eGFP-Syntaxin(TMD) or eGFP-dGryGry. These differences cannot be accounted for by differences in transgene expression levels. Rather, our data suggests that the magnitude of the variation may reflect differences in the rate of protein biosynthesis and/or membrane assembly for each transgene.

### The light-sensitive variation is tightly related to axial variation in birefringence

Previously, we have shown that Rho-eGFP has a uniform lateral distribution in disk membranes, while excluded from incisures ([30], [31]). Depending upon the light cycle, here we have observed between 30–300% variation in peak Rho-eGFP fluorescence intensity levels in dark and light. By contrast, the maximal variation in endogenous rhodopsin was <5% even in an extended light cycle. Thus, the Rho-eGFP subcellular distribution is like the endogenous rhodopsin distribution in being restricted to the OS discs but does not match the Kaplan banding in the OS. One possible explanation for this difference between native and transgenic rhodopsin is that the axial density of disk membranes along the OS may not be uniform — disks assembled in the dark may be packed more densely than those made in the light. In acutely calcium-depleted *Rana pipiens* rods, birefringence band contrast (i.e. the magnitude of the variation) increased permanently, suggesting that the lighting cycle controls a calcium-sensitive component of OS synthesis [17]. This component has not yet been identified. It could be related to the intradiscal space, which sequesters calcium [42], possibly by regulating bilayer interactions across the intradiscal space or altering molecular conformations that influence disk density. In electron micrographs of transgenic rods (Fig.1), no axial periodicity in disk membrane spacing was observed. However, the cells studied by EM were fixed in glutaraldehyde, which could have disrupted disk spacing and caused shrinkage of OS disks [17]. On the other hand, Rho-eGFP banding in fixed samples has been observed (e.g. [24] and Fig. 6), albeit with some reduction in the magnitude of the variation. Therefore, it seems unlikely that axial packing density variation of disks is responsible for the banding of rhodopsin fusion proteins.

### The light-dependent axial variation may be a consequence of light-dependent rates of disc membrane synthesis

Alternatively, the Kaplan banding of Rho-eGFP could arise from periodic variation in Rho-eGFP density in disks made during different phases of the light cycle. If so, this represents a significant difference between rhodopsin fusion proteins expressed from transgenes and endogenous rhodopsin, the basis for which is not clear. We were not able to rule out transcriptional variation of Rho-eGFP transgenes over the course of the light cycle. However, since periodic axial variation of Rho-eGFP with a spatial period of 1–1.5 µm and similar amplitudes was observed in OS from animals harboring transgenes using different promoters and reliably in many different individual F0 transgenic individuals, it seems unlikely the cause of periodic variation is predominantly at the level of transcription. Moreover, there was extremely low or undetectable periodic axial variation in OS from animals expressing eGFP-Syntaxin(TMD) driven by the same promoter, XOP, as Rho-eGFP. Thus, if Rho-eGFP distribution reflects variation in Rho-eGFP content, then we conclude that there is a light-regulated variation in the assembly rate of Rho-eGFP into disk membranes.

**Figure 11:**
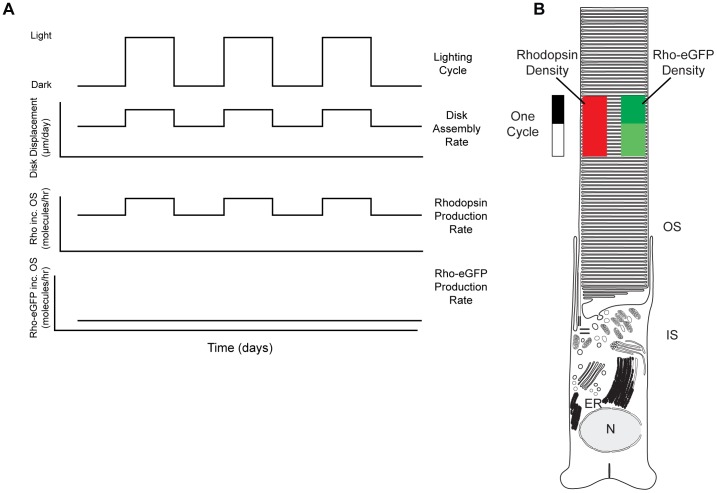
Schematic diagram of light-regulated disk assembly. A. Rods housed in an alternating light-dark cycle (*top panel*) will vary the rate of disk assembly and displacement (*second panel*) in response to the phase of the cycle [6], [7]. Since endogenous rhodopsin density in OS membranes is constant, the rate of rhodopsin incorporation (*third panel*) must change in phase with the lighting cycle and disk assembly. By contrast, Rho-eGFP appears to have a relatively constant rate of incorporation into the OS (*bottom panel*). This would then lead to a periodic variation in Rho-eGFP content, with higher densities assembled at night. B. Schematic diagram illustrating the density variation of Rho-eGFP (*green*) throughout a light cycle and the absence of variation in endogenous rhodopsin (red). The length of the lighting cycle and the size of the cell are not to scale.

Endogenous rhodopsin and Rho-eGFP are expressed at vastly different levels [30], [31]. Furthermore, endogenous rhodopsin gene does not exhibit significant light-sensitive variation in transcription (Fig. 8) and others have shown that rhodopsin translation rates do not change in response to changes in light [9]. One possible explanation for this difference could be that the rate of Rho-eGFP production is some how limited and cannot increase in the light as endogenous rhodopsin (Fig. 11A). Thus, as disk synthesis increase, Rho-eGFP density would decrease compared to those made in the dark leading to two regions of different density (Fig. 11B). Rhodopsin, on the other hand, can increase production in the light and this maintains a constant density throughout the cycle.

In amphibians, the lighting cycle has a profound effect on the rate of disk assembly and OS elongation [7]–[9], [34], [43]. Light onset triggers the assembly of disks at the base of the OS [34], [43]. In 24 h (12L:12DL) light cycles, *Xenopus* rods produce 29–91 disks per day, depending on the temperature [10]. The rate of disk displacement is 1.2–1.8 times greater in the light than in the dark, again depending upon the temperature [7]. The relative widths of the Rho-eGFP bands synthesized in the light compared to those in the dark fit within this range. Thus, it appears that rhodopsin fusion proteins are responding to the same light signals as those that regulate disk assembly and displacement.

It is not obvious why the Rho-eGFP or Rho-mCherry fusion proteins should behave differently than endogenous rhodopsin. Although eGFP and mCherry are fused to the carboxyl terminus in a flexible region of rhodopsin [44] and are synthesized after rhodopsin translation and membrane insertion is complete, it is possible that they somehow alter folding, processing or transport to the OS, thus potentially reducing the rate of incorporation of Rho-eGFP into the OS compared to endogenous rhodopsin. Since we do not observe much Rho-eGFP in the IS (also see [31]), there would have to be significant degradation of the fusion protein within the first hour post synthesis, since the eGFP maturation time is approximately one hour at these temperatures [45]. Moreover, since endogenous rhodopsin is found at uniform densities throughout the OS even in extended lighting cycles (Fig. 6 and 7), the rate of endogenous rhodopsin synthesis, trafficking (through the secretory pathway) and assembly into disks is closely matched to the light-dependent rate of disc membrane synthesis. Thus, when the latter is sped up, the incorporation of rhodopsin matches. One possibility is that the rhodopsin synthesis rate varies, and that this leads to more membrane transport and higher rate of disc synthesis in darkness. However, rhodopsin synthesis measured in retinal explants for 4 hours in the light and dark were very similar [9]. This suggests other steps may be regulated by the light cycle. Apparently rhodopsin fusion proteins are unable to adjust the rate of assembly in response to light.

### Possible explanations of the rhodopsin fusion protein light-dependent variation

There are a number of possible ways eGFP or mCherry could limit the rate of processing or assembly into the OS. The carboxyl terminus is also involved in sorting and transport of rhodopsin to the OS (reviewed in [46]). eGFP or mCherry could disturb processing through the ER and Golgi or its interaction with components of the vesicular transport machinery, leading to degradation. Recently, rhodopsin's carboxyl terminus has been implicated in rhodopsin dimer formation in membranes [47]. Thus, it is possible that the oligomeric state of Rho-eGFP may be different than endogenous rhodopsin, again potentially leading to an alteration of the rate of rhodopsin fusion proteins into disk membranes. Interestingly, an eGFP fusion protein containing the last 44 amino acids of rhodopsin at its carboxyl terminus was found in the OS, exhibiting a banding pattern similar to the periodic axial variation seen with rhodopsin transgenes [25]. However, it is not possible to quantitatively compare those images of fixed tissue with the live cell imaging. So, although the carboxyl terminus has an important role in banding, we cannot determine how important other regions of rhodopsin are for periodic axial variation. This highlights a potential complication for studies utilizing rhodopsin with carboxyl terminal alterations.

The banding observed with an arrestin-eGFP transgene does not appear to be explained by OS regions with different contents of endogenous rhodopsin. The correspondence of the arrestin-eGFP banding in light-adapted OS and the previously characterized birefringence banding suggests that there is an inhomogeneity in membrane composition or properties along the rod OS that may have physiological implications. For example, it is clear that cholesterol content changes with axial position [48]. So, it is possible that the disk assembly pathways operating in the dark or light could produce membranes with different lipid compositions potentially leading to alterations in how arrestin-eGFP interacts with rhodopsin or the membrane itself.

### Light/dark variation in disc membrane synthesis may contribute to stable OS

The stability of the OS is an important factor in photoreceptor health and survival. We recently presented a theoretical model for the mechanical properties of the OS and derived parameters regulating their flexural rigidity [49]. Axial density variations similar to the banding considered here were an integral part of the model. Calculations showed that such density variations, which contribute to OS stiffness, increase the flexibility of the OS to bending and makes it less fragile. Thus, the axial banding may have evolved to improve the mechanical properties of OS, permitting larger and longer OS containing higher amounts of rhodopsin. Furthermore, OS were predicted to have a tendency to break in higher density regions. This could have implications for disk shedding at the apical surface. In many species, there is a strong light-dependence to shedding, with most of the disks being shed soon after light onset in large packets [7] reminiscent of the bands examined here. In addition, both disk shedding and axial variation are reduced in constant light. Thus, if OS disks are shed in packets that coincide with periodic Rho-eGFP bands, then Kaplan banding may provide a means to determine how many disks will be shed or to organize and facilitate membrane breakdown. Future experiments on the structural basis of Kaplan banding will be required to determine its potential role in OS renewal.

## Methods

### Animal Husbandry

Care and feeding (three times per week) of *Xenopus* have been described in detail (Solessio et al., 2009). Animals were housed at 18–20°C in a 24 h (12L:12D) lighting cycle unless otherwise stated. All animal handling and experiments were in agreement with the animal care and use guidelines at Association for Research in Vision and Ophthalmology (ARVO). This study was done under the approval of the SUNY Upstate Medical University Committee on the Human Use of Animals (CHUA no. 209).

### Transgenic Constructs

Plasmids for transgene expression in *Xenopus* were based upon the pEGFP-N1 (Stratagene) backbone as previously described [22]. DNA fragments from *Xenopus* opsin (545 or 5417 bp) [22], *Xenopus* arrestin (XAR7, 285 bp) [50] or *Xenopus* α-transducin upstream (4996 bp) sequences (unpublished data) were generated by PCR and subcloned into the pEGFP(-) vector [22] at the XhoI-BamHI site. Dual transgene expression constructs were made by assembling each transgene protein cassette separately by PCR and then sub-cloning both into a vector containing duplicate XOP(-504/+41) promoters [50] in the same transcription direction. All protein coding sequences were sequenced on both strands prior to transgenesis.

### Transgenic *Xenopus*

Transgenic animals were produced using restriction enzyme-mediated integration (REMI) with some modifications as described [51]. Plasmids containing transgenes were linearized outside the transcription unit with either XhoI or NheI (New England Biolabs), purified (PureLink™, Invitrogen,Carlsbad, CA) and then used in the REMI reaction with the same enzyme at 0.5 U per reaction. Embryos were kept in 0.1X MMR (concentrations in mM, NaCl, 10; KCl, 2; MgSO_4_, 1; CaCl_2_, 2; HEPES, 5 at pH = 7.4) for 6 days at 16°C with a 12/12 light/dark cycle and then at 20°C.Transgenic tadpoles were selected by observing whole eye fluorescence using a fluorescence dissecting microscope on day 6 after nuclei injection and again on day 20.

### Live Cell Imaging

The recording chamber was fabricated in the center of a 5 cm plastic petri dish with a No. 1 coverslip forming the chamber bottom, allowing access of the microscope lenses as described previously [21], [31]. Pieces of retina (chips) were minced into small pieces and placed into the imaging chamber with Ringer's solution (in mM: NaCl 111, KCl 2, CaCl_2_ 1, MgCl_2_ 1, MgSO_4_ 0.5, NaH_2_PO_4_0.5, HEPES 3, glucose 10, EDTA 0.01). The chamber was covered by a No. 1 coverslip and placed onto the microscope stage for imaging. All imaging was performed at 20°C.Imaging was performed using LSM-510 (Zeiss) equipped with an Argon laser generating 488 nm laser line and a HeNe 543 laser. To reduce contamination signals across the two fluorescent channels, 500–535 nm and 655–710 nm bandpass filters were used to filter fluorescence excited by Ar and HeNe lasers respectively. The scanning objective for both channels was a Plan-Neofluor 63x/1.4 N.A. oil lens (Zeiss). The scan settings were: resolution, 0.04×0.04 µm in the *xy* plane, pixel time 3 µs, pinhole diameter 1.4 Airy units, amplifier offset 0.1 and amplifier gain 1. At least 5 *z* scans from the central area of each photoreceptor using a 0.5 µm interval were obtained. The LSM-510 software was set to correct for the *z*-plane while scanning dual channels. Intensities of images were measured using AxioVision™ software version 4.7 (Zeiss), corrected for the background intensity, and averaged values of at least 3 *z* sections were used for fluorescence intensity measurements. Intensities were collected in arbitrary units (0–255) and then were constrained normalized to 0-100.The magnitude of the density variation in OS bands was estimated as follows. Images were selected, and four to five central *z* planes were deconvolved using theoretical mode in Axiovision 4.7 deconvolution module (Zeiss). For a given cell, three consecutive bands with similar amplitudes were selected and used to determine the average ratio of maximum to minimum density for those bands. Ratios were determined for more than 90 bands from 10 cells, and then averaged to calculate the fluorescence intensity differences between the maximum and minimum levels in a band.

### FRAP analysis by confocal microscopy

The recording chamber was initially searched for retinal chips with the rod axis parallel to the coverslip. A single rod photoreceptor was then centered in the imaging window and 8 bit images in gray scale were scanned from a 13.2×13.2 µm in *xy* plane (256×256 pixel). Photobleaching was performed in a 1.5 µm-wide rectangular in the central region of the rod OS for 40 ms with the 488 nm laser line. Images were acquired before and after photobleaching. All laser scanning and bleaching was at the same *z* axis. The images in the recovery phase were taken immediately after photobleaching and then every 3–5 s in time series of at least 25 recovery scans.

### Immunohistochemistry and electron microscopy

For immunohistochemistry, eyes were fixed in 4% paraformaldehyde in PBS at 4°C and frozen in OCT (TissueTek Inc.). Frozen blocks were sectioned into 12–20 µm slices with a Microm560 cryostat (Richard Allen Scientific). The tissue on slide was permeabilized with 0.5% Triton X-100 in 1X PBS at room temperature, blocked with5% goat serum, 0.1% Triton X-100 in 1X PBS at 4°Cand immunostained with the primary antibody in blocking buffer for 96 hrs in a humidified chamber at room temperature, washed and then incubated with appropriate conjugated secondary antibody, and finally, washed and mounted. Primary antibodies used were mouse anti-rhodopsin K16-155C (C-terminus, 1∶100) mouse anti-rhodopsin 4D2 (N-terminus, 1∶20,000), mouse anti-rhodopsin 1D4 (C-terminus, 1∶1000), and anti-mouse secondary antibody (Jackson ImmunoResearch) conjugated to Cy3 (1∶750). Slides were examined with either a C2 series (Nikon) or LSM-510 confocal microscope (Zeiss). For preparation of EM sections, eyes were fixed in 2.5% glutaraldehyde and 1% OsO_4_ in phosphate buffer and embedded in epoxy resin as described previously [52].

### Microspectroscopy

Rods were obtained from animals housed in a 168 h (84L–84D) light cycle at 20–22°C. Rhodopsin density was determined by measuring the density before and after bleaching the rod using a microdensitometer with submicrometer spatial resolution as previously described [30].

### Quantitative real-time PCR

To study the variation of light-sensitive gene expression, male *Xenopus* (8–10 cm long) were kept at 25°C with a 24 h (10D/14L) light cycle for at least 2 weeks prior to any experiment or in constant conditions for 24 h. Animals were sacrificed at 4 h intervals, three pairs of retina were separated in the dark from the RP, pooled and stored at −80°C. Total RNA was isolated from one (examination of endogenous rhodopsin in extended 24 h light or dark period) or three pairs (examination of genes every 4 h) of retinas using RNeasy kit (Qiagen) at each time point. RNA integrity was verified using a Bioanalyzer (Agilent 2100). cDNA was synthesized using QuantiTect Reverse Transcription Kit (Qiagen). Primers were designed using Primer3 (http://frodo.wi.mit.edu/): β-actin, 5′GCACCCCTGAATCCTAAAGC3′ and 5′TTGGCACAGTGTGGGTTACA3′; EF1-α, 5′GATTGATCGCCGTTCTGGTA3′ and 5′GCTTTCCTGGGATCATGTCA3′; rhodopsin, 5′ATGACCGTCCCAGCTTTCTT 3′ and 5′CACCTGGCTGGAAGAGACAG3′; red cone opsin, 5′TCTTTGCCTGTTTTGCTGCT3′ and 5′TCCATCATCGACCTTTTTGC3′; nocturnin, 5′GCTGTGCCTTGTTCTTCCTG3′ and 5′TTAGATGGGTGACCGCAAAG3′). The accession numbers for the *Xenopus* genes used in this study are: L07770 (rhodopsin), BC081156 (red cone opsin), U74761 (nocturnin), AF079161 (β-actin), X02995 (18S ribosomal RNA), NM_001087442 (EF1α), X03017 (histone 4, [53]). Real-time PCR (cDNA from 5 ng reverse transcribed total RNA and 2.5 µM of each primer mix containing SYBR Green I) was performed using Roche LightCycler 480 (Roche Diagnostic). PCR amplification was performed following denaturation (95°C for 10 min) for 45 cycles of 95°C for 15 s, 60°C for 15 s, 72°C for 30 s, 80°C for 5 s. Finally, a product melting curve was obtained by heating the samples from 60–95°C (heating rate of 0.1°C per second) with continuous fluorescence measurement, followed by a cooling step to 25°C.RT PCR data analysis was performed as described [54]. The crossing point (CP) was determined using LightCycler software 3.3 (Roche Diagnostics) for each primer-pair PCR reaction. To determine the relative expression of a gene at different times of day, the difference between the CP value for a particular time (T) was subtracted from a standard time point, chosen arbitrarily as 8 AM. This difference was termed ΔCP = CP^T^-CP^8 AM^. To normalize for potential variation in tissue harvesting or RNA processing, comparisons of ΔCP from target genes were referenced to β-actin. Similar results were obtained when target genes were referenced to EF1α (*not shown*). The PCR amplification efficiencies (*E*_target_ and E_βactin_), were determined from the slope of the real time PCR curves based on the relationship, E = 10^[-1/slope]^ [55]. Thus, the relative RNA abundance at time T (R^T^) for a target RNA compared to β-actin RNA was estimated using the following formula: Relative Expression = (EΔ^CP^_target_^)(t^_8AM_^-t)^)/(^EΔCP^_actin_^)(t^_8AM_^-t)^). For each time point, retina from three animals were collected, pooled and analyzed by real-time PCR. In addition, each time point was repeated with a separate group of animals sacrificed on different days. R^T^ from the two groups at each time was averaged (with standard deviation). One-way analysis of variance (ANOVA) between the different time points was used to determine significance of differences across the light cycle. Statistics on relative expression of endogenous rhodopsin, nocturnin, and red cone opsin after normalization to β-actin are: Rho F(6, 7)  = 0.54, p = 0.7630; nocturnin F(6, 7)  = 12.41, p = 0.0020; red cone opsin F(6, 7)  = 6.34, p = 0.0141.

## Supporting Information

Figure S1 **Axial variation in cells expressing either soluble eGFP (A) or Rho-eGFP (B) transgenes.** (A) eGFP fluorescence is found in both IS and OS and does not exhibit periodic axial variation. The intensity profile of the fluorescence along the OS axis (*white line*) is shown below. (B) Some rods expressing the Rho-eGFP transgene exhibit wide variation in fluorescence intensity along with a superimposed periodic axial variation with a spatial period of ∼1.5 µm. The intensity profile of the fluorescence along the OS axis (white line) is shown below. Animals were housed in a 24 h (12D:12L) cycle. Compare the average fluorescence intensity in the region between 0–22 µm to that in the region between 25–45 µm. This arises from mosaic transgene expression.
(TIF)

Figure S2 **Axial variation in cells expressing Rho-eGFP.** A. Selected cells expressing Rho-eGFP under control of a *Xenopus* opsin promoter (∼0.6 kb) from four different transgenic lines (F_1_) housed in the same 24 h (12D:12L) cycle exhibit in-phase axial banding but asynchronous slower variation. Scale bar, 10 µm. (B–E) To characterize the asynchronous temporal variation, the fluorescence intensity profile (red circles) from the 20–30 µm proximal to the OS base was fit to a sinusoidal function (black lines) using SigmaPlot12 (Jandel Scientific). (F–G) A box plot (F) and histogram show the spatial period (µm) of the best fit sinusoidal function for each of the cells in (A).There was a range of frequencies across the cells, with a median of 27 µm corresponding to temporal frequency of ∼18 days.
(TIF)

Figure S3 **Axial variation in OS fluorescence in rods expressing of Rho-eGFP and Rho-mCherry under the control of rod-specific promoters.** Top Right, a schematic diagram of plasmid constructs (#1–#8, *see Methods for details*) used for transgenesis in this study is shown. Representative images of OS fluorescence from rods harboring transgenes that expresses Rho-eGFP and/or Rho-mCherry under control of a various promoters are shown. (A) A dual transgene that expresses Rho-eGFP and Rho-mCherry under control of a *Xenopus* opsin promoter (∼0.6 kb) from the same locus. The axial variation is precisely in phase while the asynchronous variation is less tightly coupled. (B) A dual transgene that expresses Rho-eGFP under control of a *Xenopus* opsin short promoter (∼0.6 kb) and Rho-mCherry under the control of a *Xenopus* opsin long promoter (∼5.5 kb). (C) A transgene that expresses Rho-eGFP under control of a *Xenopus* arrestin promoter. (D) A transgene that expresses Rho-eGFP under control of a *Xenopus* rod transducin promoter. (E) A dual transgene that expresses Rho-eGFP under the control of a *Xenopus* arrestin promoter and Rho-mCherry under control of a short Xenopus opsin promoter. Scale bar, 5 µm.
(TIF)
