# Supplementary figures and images for: Regulation of Rhodopsin-eGFP Distribution in Transgenic Xenopus Rod Outer Segments by Light
Source: PLoS One. 2013 Nov 15;8(11):e80059. doi: 10.1371/journal.pone.0080059 (PMC3829889; doi:10.1371/journal.pone.0080059)

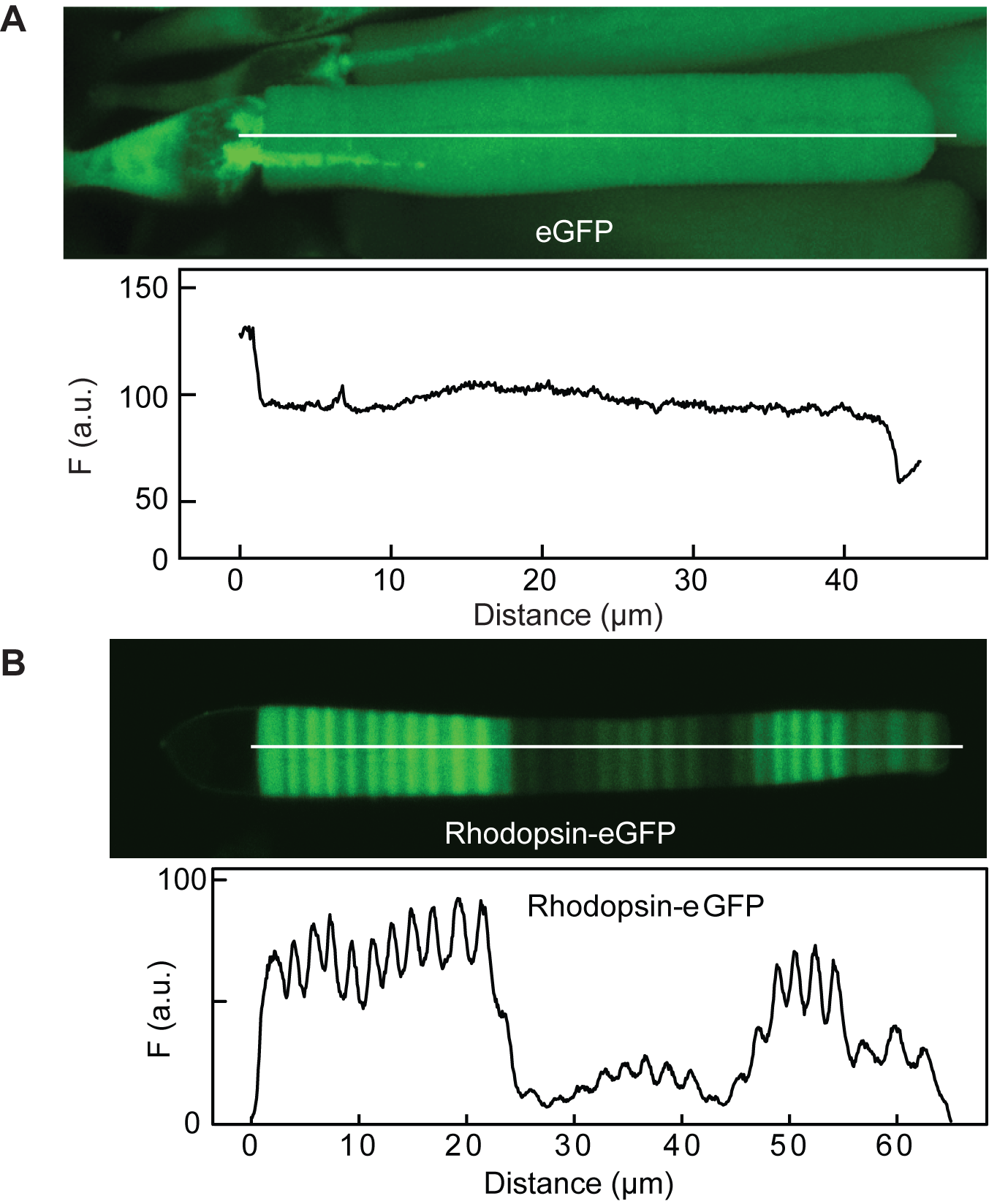

Supplement: Figure S1 — Axial variation in cells expressing either soluble eGFP (A) or Rho-eGFP (B) transgenes. (A) eGFP fluorescence is found in both IS and OS and does not exhibit periodic axial variation. The intensity profile of the fluorescence along the OS axis (white line) is shown below. (B) Some rods expressing the Rho-eGFP transgene exhibit wide variation in fluorescence intensity along with a superimposed periodic axial variation with a spatial period of ∼1.5 µm. The intensity profile of the fluorescence along the OS axis (white line) is shown below. Animals were housed in a 24 h (12D:12L) cycle. Compare the average fluorescence intensity in the region between 0–22 µm to that in the region between 25–45 µm. This arises from mosaic transgene expression. (TIF) [file pone.0080059.s001.tif]

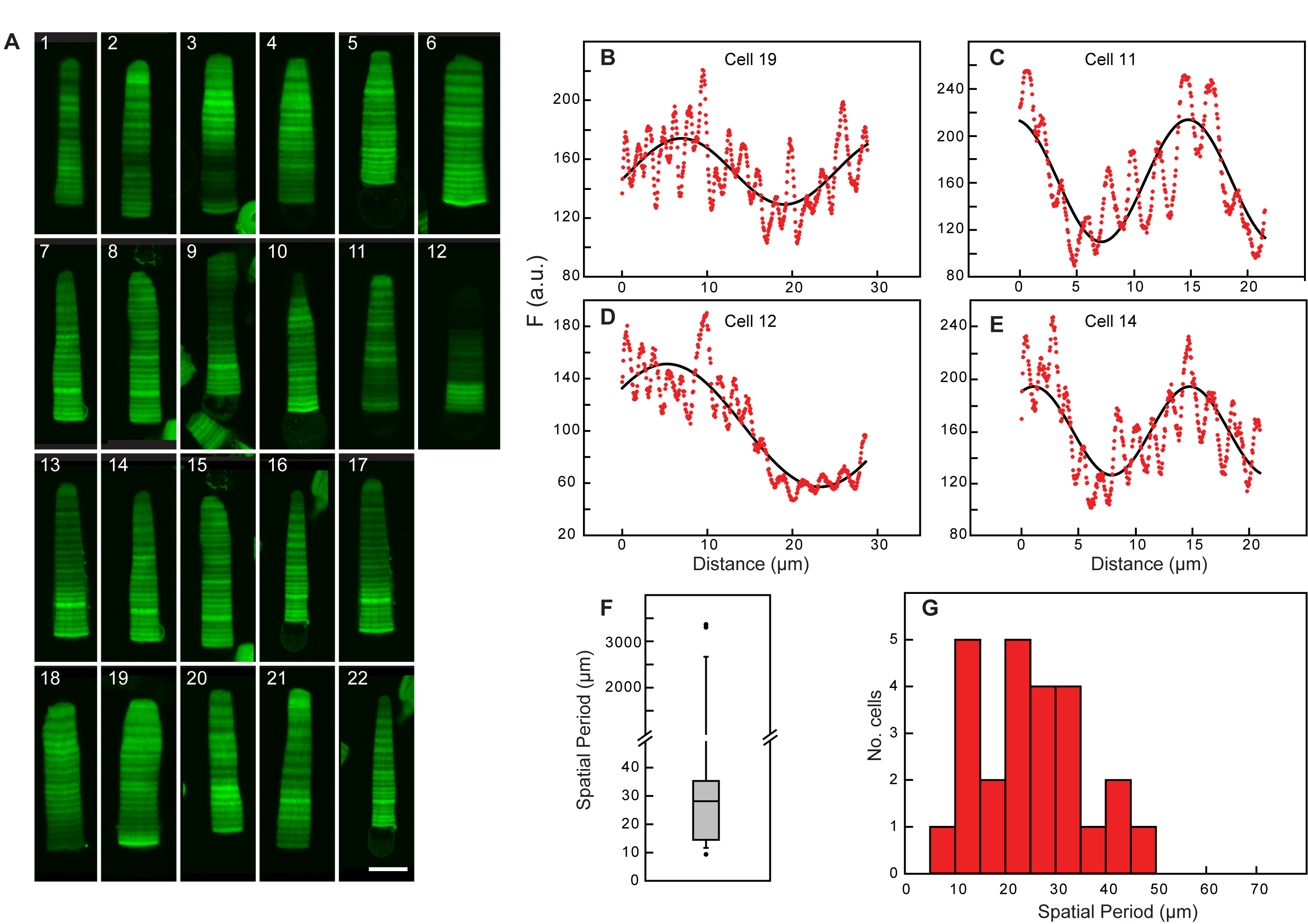

Supplement: Figure S2 — Axial variation in cells expressing Rho-eGFP. A. Selected cells expressing Rho-eGFP under control of a Xenopus opsin promoter (∼0.6 kb) from four different transgenic lines (F1) housed in the same 24 h (12D:12L) cycle exhibit in-phase axial banding but asynchronous slower variation. Scale bar, 10 µm. (B–E) To characterize the asynchronous temporal variation, the fluorescence intensity profile (red circles) from the 20–30 µm proximal to the OS base was fit to a sinusoidal function (black lines) using SigmaPlot12 (Jandel Scientific). (F–G) A box plot (F) and histogram show the spatial period (µm) of the best fit sinusoidal function for each of the cells in (A).There was a range of frequencies across the cells, with a median of 27 µm corresponding to temporal frequency of ∼18 days. (TIF) [file pone.0080059.s002.tif]

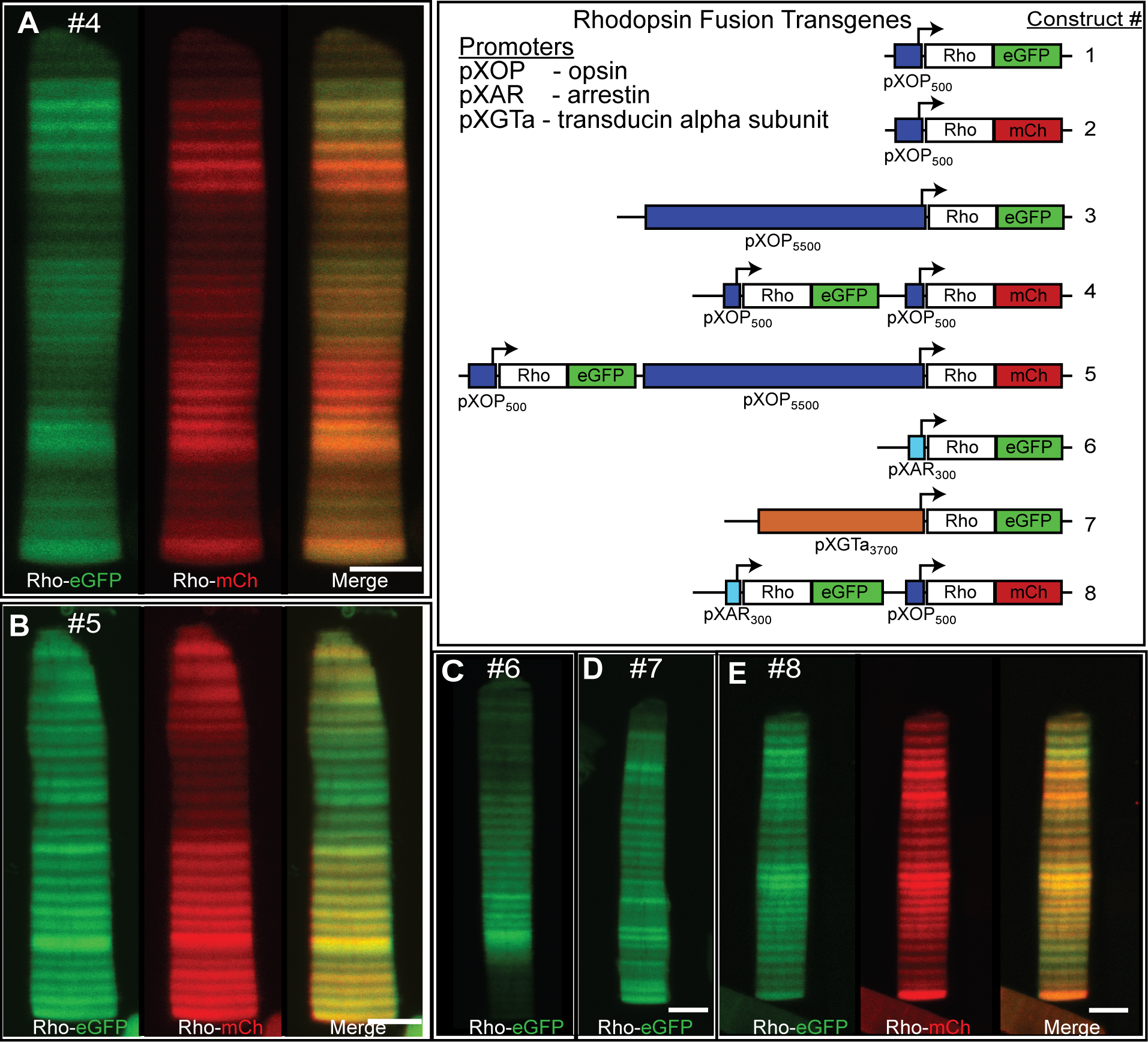

Supplement: Figure S3 — Axial variation in OS fluorescence in rods expressing of Rho-eGFP and Rho-mCherry under the control of rod-specific promoters. Top Right, a schematic diagram of plasmid constructs (#1–#8, see Methods for details) used for transgenesis in this study is shown. Representative images of OS fluorescence from rods harboring transgenes that expresses Rho-eGFP and/or Rho-mCherry under control of a various promoters are shown. (A) A dual transgene that expresses Rho-eGFP and Rho-mCherry under control of a Xenopus opsin promoter (∼0.6 kb) from the same locus. The axial variation is precisely in phase while the asynchronous variation is less tightly coupled. (B) A dual transgene that expresses Rho-eGFP under control of a Xenopus opsin short promoter (∼0.6 kb) and Rho-mCherry under the control of a Xenopus opsin long promoter (∼5.5 kb). (C) A transgene that expresses Rho-eGFP under control of a Xenopus arrestin promoter. (D) A transgene that expresses Rho-eGFP under control of a Xenopus rod transducin promoter. (E) A dual transgene that expresses Rho-eGFP under the control of a Xenopus arrestin promoter and Rho-mCherry under control of a short Xenopus opsin promoter. Scale bar, 5 µm. (TIF) [file pone.0080059.s003.tif]
